# Highly Conductive In-SnO_2_/RGO Nano-Heterostructures with Improved Lithium-Ion Battery Performance

**DOI:** 10.1038/srep25860

**Published:** 2016-05-11

**Authors:** Ying Liu, Alessandro Palmieri, Junkai He, Yongtao Meng, Nicole Beauregard, Steven L. Suib, William E. Mustain

**Affiliations:** 1 Department of Chemical & Biomolecular Engineering, University of Connecticut Storrs, CT 06269-3222, USA; 2 Center for Clean Energy Engineering, University of Connecticut, Storrs, CT 06269-5233, USA; 3 Institute of Materials Science, University of Connecticut, Storrs, CT 06269-3136, USA; 4 Department of Chemistry, University of Connecticut, Storrs, CT, USA.

## Abstract

The increasing demand of emerging technologies for high energy density electrochemical storage has led many researchers to look for alternative anode materials to graphite. The most promising conversion and alloying materials do not yet possess acceptable cycle life or rate capability. In this work, we use tin oxide, SnO_2_, as a representative anode material to explore the influence of graphene incorporation and In-doping to increase the electronic conductivity and concomitantly improve capacity retention and cycle life. It was found that the incorporation of In into SnO_2_ reduces the charge transfer resistance during cycling, prolonging life. It is also hypothesized that the increased conductivity allows the tin oxide conversion and alloying reactions to both be reversible, leading to very high capacity near 1200 mAh/g. Finally, the electrodes show excellent rate capability with a capacity of over 200 mAh/g at 10C.

Tin oxide has been extensively explored recently as a high energy density alternative anode material to traditional graphite. Some work has focused on the reaction of tin with lithium to form various Li_x_Sn reversible alloys with a theoretical capacity as high as 993 mAh/g, more than two and a half times that of graphite (372 mAh/g)^1^. However, two key issues hindering the commercialization of tin oxide remain unsolved: poor conductivity retention and large volume expansion. It is known that the formation of Li_x_Sn alloys involves up to five different crystallographic phases with x ranging from 0 to 4.4, resulting in volume changes as large as 260%. The coexisting alloy phases, combined with uneven Li concentrations, can lead to the inhomogeneous volume expansions of tin oxide and even anode cracking or pulverization, as well as the loss of electrical contact, which can be detrimental to its long-term performance.

To circumvent these challenges, the use of a highly conductive matrix and nanostructured metal oxides can be efficient methods to improve the cycling stability of metal oxides by suppressing their volume change and increasing their electrical conductivity^2^,^3^,^4^,^5^,^6^. Graphene, with intrinsically excellent electrical conductivity and mechanical flexibility, has been proposed as one of the most appealing carbon materials for this purpose. Graphene also has the advantage of being non-dilutive because, unlike common carbon additive materials, it has the ability to store charge^7^. A variety of metal oxides with different sizes and morphologies have been deposited on graphene as anode materials for LIBs and have shown improved capacity, rate capability, and cycling stability^8^,^9^. The ultrathin flexible graphene layers can provide a support for anchoring well-dispersed nanoparticles (NPs), which can effectively prevent global volume expansion/contraction and aggregation of NPs during the Li charge/discharge process. They also work as a highly conductive matrix for efficient electron transport. Meanwhile, the anchoring of NPs on graphene can effectively reduce the degree of restacking of graphene sheets and consequently keep their high active surface area and to some extent can increase the lithium storage capacity and cyclic performance. It is well-accepted that nanomaterials have advantages of good cycling performance and short path length for Li^+^ transport over their bulk counterparts. Therefore, it is believed that the composite of flexible and electrically conductive graphene anchored with nanostructured SnO_2_ particles can lead to LIBs with superior performance.

Though graphene is excellent for providing external electron transport pathways for SnO_2,_ another way to modify material properties is to introduce dopants, like indium, into the SnO_2_ structure for internal conductivity improvement, because indium doped tin oxide (ITO) is known to have much higher conductivity compared with semiconductor SnO_2._ Herein, we report a facile strategy to synthesize such composite In-SnO_2_ (ITO) NPs anchored onto conducting graphene as an advanced anode material for high-performance LIBs. The ITO NPs obtained are around 20 nm in size and are homogeneously anchored on the graphene sheets as spacers to keep the neighboring sheets separated. This ITO/RGO nanocomposite displays superior LIB performance with large reversible capacity, much higher than bare SnO_2_ or In_2_O_3_ (289 mAh/g), high Coulombic efficiency, excellent cycling performance, and good rate capability-highlighting the potential importance of dopants and NP anchoring on graphene sheets for achieving high-performance LIB anodes.

## Results

**Figure 1:**
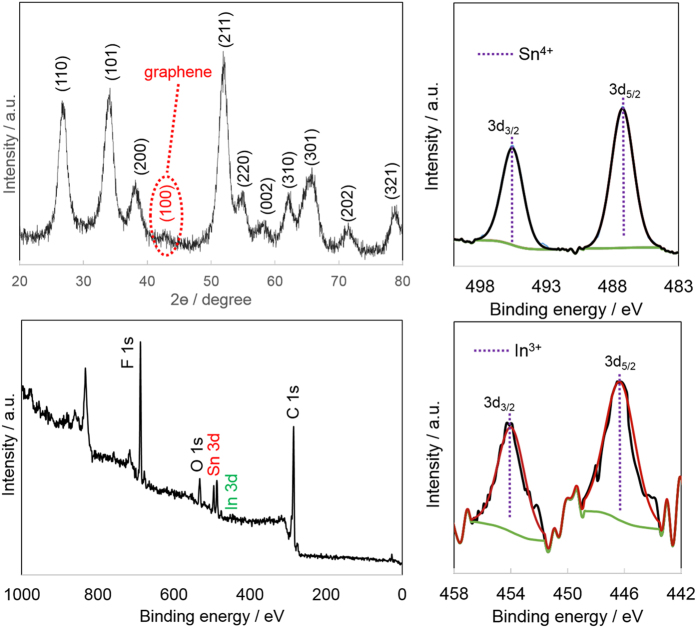
(**a**) Powder XRD pattern of ITO/RGO composite, XPS spectrum of ITO/RGO composite, (**b**) broad scan spectra, high resolution spectra of (**c**) Sn 3d and (**d**) In 3d.

Figure 1 shows a typical powder X-ray diffraction (XRD) pattern for the synthesized ITO/RGO composite anode material. Compared to pure ITO (Fig. S1, Supporting information), an additional low intensity, broad (100) diffraction peak appeared at 2Θ = 43.5°, which can be indexed to disorderedly stacked graphene sheets (Fig. S2), though this broad peak is weaker than that of the as-prepared graphene. All of the other diffraction peaks can be ascribed to a rutile SnO_2_ structure (JCPDS 041-1445) with no secondary phases, indicating the effective incorporation of In into the SnO_2_. The 10 at% In in SnO_2_ was confirmed by EDS. The XRD patterns suggest that the composite consists of stacked RGO sheets and well-crystallized ITO. The XRD pattern of In_2_O_3_/RGO (Fig. S3) shows a bixbyite In_2_O_3_ cubic structure (JCPDS 06-0416) and a similar characteristic (100) graphene peak^10^.

X-ray photoelectron spectroscopy (XPS) was used to identify the oxidation and coordination states of the surface In and Sn of the ITO/RGO composite. The general spectra was collected from 0-1100 eV region (Fig. 1b). XPS identified O, F, C, Sn and In elements, and the In/Sn ratio was 0.045, almost half of the bulk composition, which should be due to Sn surface segregation from the bulk ITO structure^11^. The Sn high resolution XPS spectra is shown in Fig. 1c. The Sn spectra could only be deconvoluted into one doublet corresponding to SnO_2_ (two peaks) with binding energies of 486.2 eV and 495.4 eV for Sn 3d_5/2_ and 3d_3/2_, respectively^12^. Similarly, only one pair of peaks was obtained for the In 3d spectra of ITO/RGO, at 446.2 and 454.1 for In 3d_5/2_ and 3d_3/2,_ which corresponds to In_2_O_3_.

**Figure 2:**
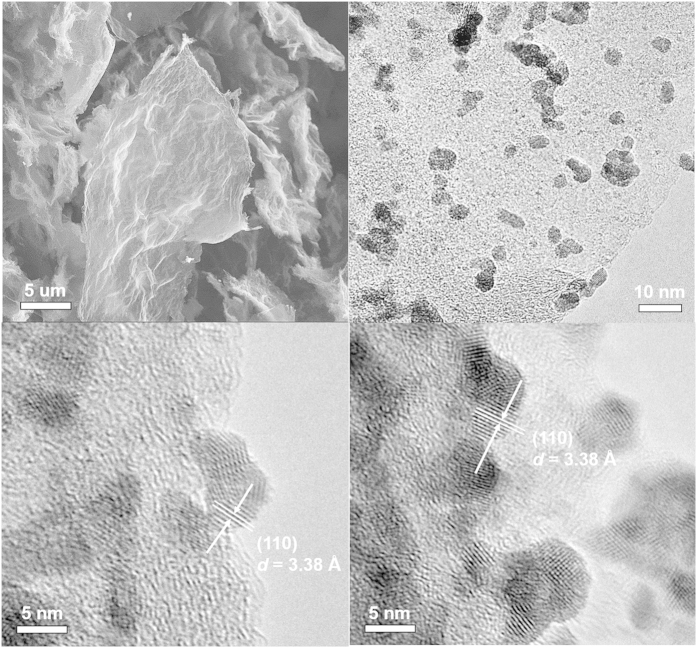
(**a**) SEM image of graphene. Low-magnification TEM image (**b**) and HRTEM images (**c**,**d**) of ITO/RGO.

SEM images of the as-prepared graphene (Figs 2a and S5a) showed a sheet-like structure. SEM and HRTEM images of the as-prepared ITO/RGO composites (Fig. 2b and Fig. S5b,c) showed that the ITO nanocrystals were homogeneously distributed on the graphene sheets, while most of the graphene sheets were packed and not fully exfoliated. HRTEM images of the ITO/RGO (Fig. 2c,d) show that the fully crystalized ITO nanocrystals possessed a lattice spacing of 3.38 Å between adjacent lattice planes, corresponding to the typical SnO_2_ (110) crystal planes; in addition, ITO nanocrystals were firmly anchored on the surface of the graphene sheets, and the presence of graphene plays an essential role for the homogeneous dispersion of ITO nanocrystals.

**Figure 3:**
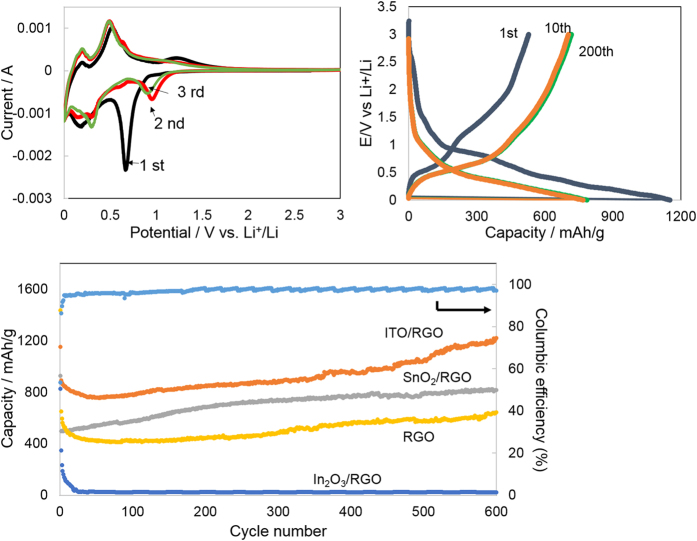
(**a**) Cyclic voltammograms of the ITO/graphene composite between 0.01 and 3 V vs. Li^+^/Li at a scan rate of 0.1 mV/s. (**b**) Galvanostatic charge/discharge profiles of the ITO/RGO cycled at 1^st^, 10^th^, 200^th^ between 0.01 and 3 V (vs. Li+/Li) at 1C current density. (**c**) Cycling performance of ITO/RGO, SnO_2_/RGO, In_2_O_3_/RGO and RGO electrodes and Columbic efficiency of ITO/RGO electrode.

Figure 3a presents cyclic voltammograms (CV) of the first three cycles of the as-prepared ITO/RGO between 0.001 and 3 V vs Li^+^/Li with a scan rate of 0.1 mV/s. In the first cycle, one large cathodic peak was observed at 0.75 V, which corresponded to electrolyte decomposition and the formation of the SEI. Also overlapping the SEI peak was the conversion of SnO_2_ to metallic Sn and Li_2_O, as described in Equation (1)^13^,^14^.


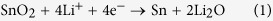


Another clear peak was observed around 0.19 V, corresponding to the formation of a Li_x_Sn alloy phase, Equation (2), which leads to a theoretical capacity as high as 993 mAh/g.


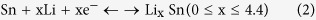


If both the conversion reaction (Equation 1) and alloying reaction (Equation 2) were simultaneously active, the maximum theoretical capacity would be 1773 mAh/g, though most studies have reported that the conversion reaction is irreversible^15^,^16^.

During the first positive scan, the peaks at 0.22 V, 0.51 V and 1.25 V can be ascribed to the phase transition from the Li_x_Sn alloy to metallic Sn, then SnO_2_ and SEI decomposition, respectively^12^,^17^. The second and third cycle CV curves almost overlap, which indicate that ITO/RGO and SEI were generally stable after the initial cycle. As shown in Fig. S6a, CVs of SnO_2_/RGO show similar peaks to ITO/RGO, while the peaks of In_2_O_3_/RGO are considerably different (Fig. S6b). For the In_2_O_3_/RGO anode, there are two cathodic peaks at 0.61 and 0.45 V, corresponding to the reduction of In_2_O_3_ to metallic indium (Equation 3) and SEI formation, and the alloying of indium with lithium^18^ (Equation 4),


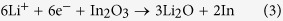



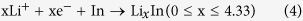


The three oxidative peaks located at 0.49, 0.65 and 1.35 V can be ascribed to the de-alloying reactions of Li_x_In alloys of various stoichiometry and the further formation of metallic phase indium and In_2_O_3_. The peak at 1.35 V fades away quickly after the first cycle suggesting that the In_2_O_3_ is irreversibly converted to In in the first cycle.

Figure 3c shows the cycling performance of ITO/RGO electrodes at 1C. The initial charge and discharge capacities of the ITO/RGO were 1058 and 563 mAh/g, respectively, based on the total mass of the ITO/RGO material, corresponding to a columbic efficiency (CE) of 53.3%. The 46.7% capacity loss of the ITO/RGO electrode can be mainly ascribed to the formation of the SEI. After the initial conditioning cycles, the CE increased to more than 96%, and the charge/discharge curves after cycle 5 all overlap (Fig. 3b). The capacity gradually increased to ~1192 mAh/g after 600 cycles. In control experiments, the SnO_2_/RGO, In_2_O_3_/RGO, graphene were prepared and tested as anodes. As shown in Fig. 3c, both SnO_2_/RGO and In_2_O_3_/RGO exhibited lower specific capacity than that of ITO/RGO. After 600 cycles, the specific charge capacity of the SnO_2_/RGO was around 2/3 that for ITO/RGO. Raw RGO, as expected, showed a stable, reversible capacity near 400 mAh/g, around ½ the capacity of ITO/RGO. In_2_O_3_/RGO completely lost its capacity after only 20 cycles, which was due to the irreversibility in forming reduced metallic indium phases inside of the SnO_2_, as indicated by XPS and In_2_O_3_/RGO CVs.

The constantly increasing capacity for RGO and ITO/RGO is likely due to the gradual exfoliation of graphene nanosheets^19^,^20^. Graphene exfoliation can improve the electronic conductivity throughout the active layer with increased cycle number, which may allow the tin oxide matrix to undergo both conversion and alloying reactions reversibly. Access to both mechanisms with increased cycle number is supported by the very high reversible capacity after 600 cycles and the ability for ITO to break through the theoretical limit for the SnO_2_ for either reaction. This suggests that the metallic In phase formed during the cycling in within the ITO/RGO structure is actually helpful for improved performance. The impact of the metallic indium will be discussed further later.

**Figure 4:**
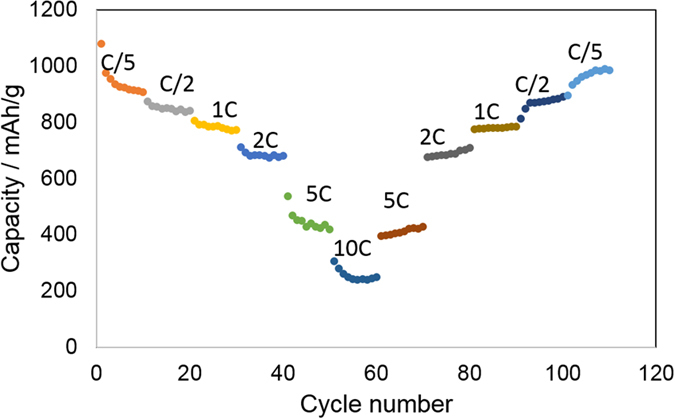
Rate capability of the ITO/RGO electrode.

The ITO/RGO electrode also showed excellent rate performance at various rates between C/5 and 10C (Fig. 4). ITO/RGO was able to maintain a stable reversible capacity over 10 cycles at each rate, and the capacity was recovered when the rate was decreased. Additionally, the high rate performance was very good; at 10C, ITO/RGO still showed a capacity over 200 mAh/g.

**Figure 5:**
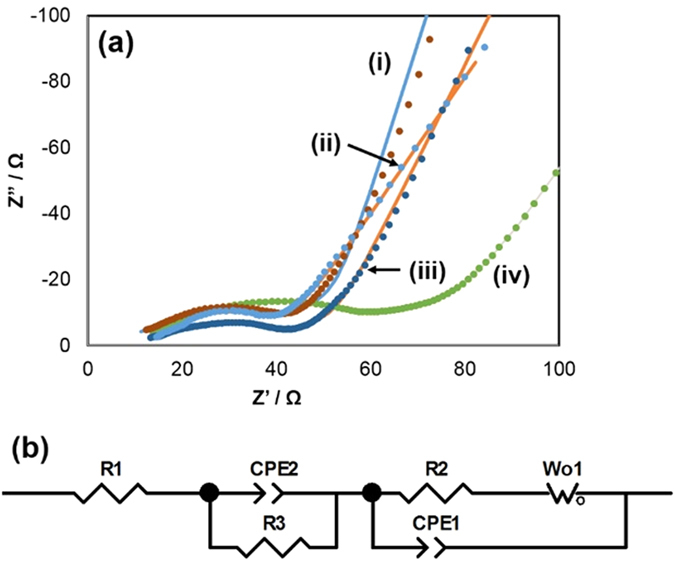
The (**a**) Nyquist plots and (**b**) corresponding equivalent electrical circuit of the ITO/RGO and SnO_2_/RGO before and after 600 cycles, (i) ITO/RGO before, (ii) ITO/RGO after, (iii) SnO_2_/RGO before and (iv) SnO_2_/RGO after.

In order to understand the effect of indium incorporation on the electrochemical behavior of the composite nanostructures, impedance measurements were carried out for the cell assembled with ITO/RGO and SnO_2_/RGO as the anode before and after 600 cycles. The Nyquist plot of ITO/RGO and SnO_2_/RGO, as well as the corresponding equivalent electrical circuit are shown in Fig. 5.

In the ITO/RGO Nyquist plot, the high frequency semicircle is related to the lithium-ion migration through the SEI film covered on ITO/graphene. The intermediate-frequency semicircle is related to the charge transfer through the electrode/electrolyte interface, and the steep sloping line at lower frequencies is related to solid-state diffusion of the lithium-ion in the electrode.

In the equivalent electrical circuit, R1 represents the total resistance of electrolyte, electrode and separator. R2 and CPE2 are the resistance and constant phase element of the SEI formed on the electrode. The middle frequency region of the Nyquist plot corresponds to charge-transfer resistance R3 and capacitance CPE3. W1 is the Warburg impedance element related to the diffusion of lithium ions into the bulk electrode. The corresponding data are shown in Table 1. It is clear that compared with SnO_2_/RGO, which showed a 60% resistance increase over 600 cycles, the total resistance of ITO/RGO after 600 cycles is almost unchanged. The result confirms that the formed metallic indium helped to preserve the high conductivity of the ITO/RGO electrode, thus significantly enhancing performance during the cycling process.

**Figure 6:**
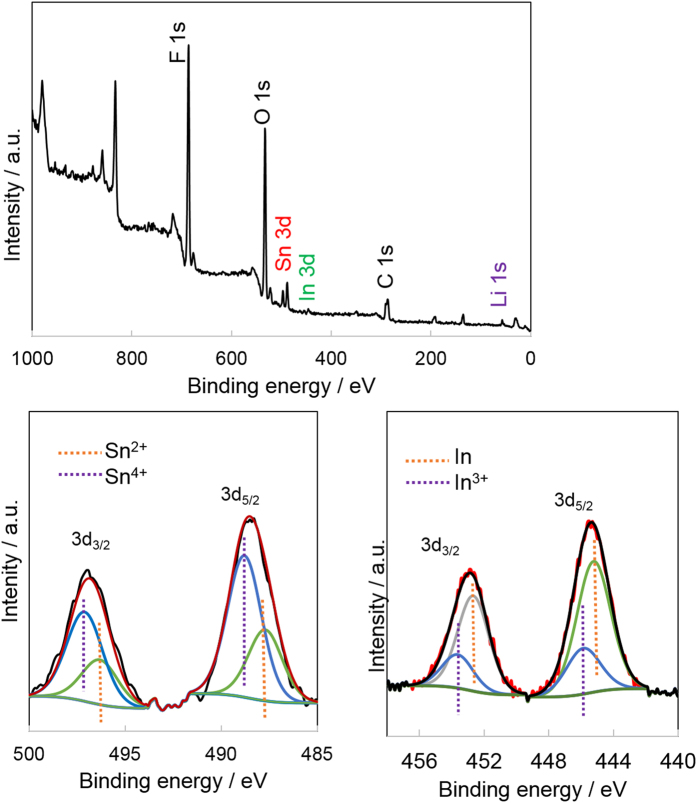
XPS spectrum of ITO/RGO composite after 600 cycles, (**a**) general spectra, high resolution spectra of (**b**) Sn 3d and (**c**) In 3d.

The XPS spectra of the ITO/RGO composite after 600 cycles are shown in Fig. 6. Both the surface In and Sn were reduced after cycling, which suggests that the first reactions between SnO_2_, In_2_O_3_ and lithium are not totally reversible, resulting in the generation of SnO and In. Compared with In, Sn showed a much better reversibility, as proved by the high Sn^4+^ to Sn^2+^ ratio, no existence of metallic Sn, in Fig. 6b. An overwhelming portion of surface In was In^0^, which further proves the existence of metallic In in ITO/RGO during cycling.

## Discussion

From the EIS and cycling experiments, the most likely cause for the high capacity of ITO/RGO was the reduced charge transfer resistance compared to SnO_2_/RGO as a function of cycle life. The metallic In nanoparticles likely act either internally to increase the electronic conductivity of the active material; they may also act as a metal-oxide bond breaking catalyst, though we did not find that adding indium significantly improved the roundtrip thermodynamic efficiency during charge and discharge.

Another important consideration is the change in the ITO/RGO structure during cycling, which was investigated by IL-TEM^21^. ITO/RGO was deposited onto a Cu TEM grid and imaged. After taking the initial TEM images, the TEM grid was placed onto a Cu disk-type electrode and held into place using a Teflon cap. The ITO/RGO coated electrode was then cycled twice and then imaged again. The structural time progression for the ITO/RGO is shown in the Supporting Information, Fig. S7. It was clear that the ITO/RGO morphology changed during cycling. The particles did not appear to agglomerate into larger clusters, but it seems that there is an extension of the graphene matrix, probably due to the progressive exfoliation of the carbon layers. The change in the diffraction pattern (Fig. S7) clearly showed that there was a complete change in the material structure, from crystalline to amorphous, which our group also saw in our previous work on NiO anodes^21^.

Metal oxides are promising due to their high capacity, relatively low cost and ease of synthesis^22^. However, mitigating capacity fade during charge-discharge resulting from agglomeration and conductivity loss is still a big issue. In this work, we developed a facile method to synthesize a composite of electrically conductive graphene anchored with ITO nanoparticles as an advanced anode material for high performance lithium-ion batteries. The ITO particles were homogeneously anchored onto graphene as spacers to keep the neighboring graphene sheets separated. Also, the strong interaction between ITO and graphene can efficiently prevent volume expansion/contraction and aggregation of ITO during the Li charge/discharge process. Moreover, metallic In was found to form and stay in the ITO/RGO structure, and combined with graphene to preserve the high conductivity of this anode material. Thus, ITO/RGO exhibits a large reversible capacity, excellent cyclability and good rate capability, and could be a promising candidate anode for next-generation lithium-ion batteries.

## Methods

### Synthesis of ITO/RGO, SnO_2_/RGO and In_2_O_3_/RGO hybrids

Graphite oxide was synthesized from graphite powder by a modified Hummers’ method. The prepared graphite oxide (20 mg) was exfoliated in distilled water (10 mL) by sonication for 60 min to form a homogeneous suspension. To prepare ITO/RGO, SnCl_2_·2H_2_O (400 mg, Sigma-Aldrich, 98%) and InCl_3_ (44 mg, Sigma-Aldrich, 98%) were dissolved in 200 ml HCl solution (0.02 M, Sigma-Aldrich, 37%). The graphite oxide suspension and SnCl_2_–HCl solution were mixed under vigorous stirring while adding 500 mg urea (Sigma-Aldrich, 98%) to form a uniform solution. The resulting solution was ultrasonicated for 30 min, then refluxed at 120 °C for 6 h. When cooled to room temperature, the solid products were washed with distilled water 6 times to remove the byproducts, followed by drying at 100 °C under vacuum overnight. The powders were further heat-treated in a tube furnace at 500 °C for 2 h under Ar flow with a heating rate of 2 °C min^−1^. SnO_2_/RGO and In_2_O_3_/RGO were prepared in the same way without the presence of indium and tin additions, respectively.

### Synthesis of raw SnO_2_, In_2_O_3_ and ITO

SnO_2_, In_2_O_3_ and ITO were also prepared in order to compare their performance to their RGO-supported counterparts. The synthesis of these materials were identical to the RGO samples simply without the presence of graphene.

### Structural characterization

The bulk structure of the synthesized materials was determined by X-ray Diffraction (XRD) with a Bruker D8 advance diffractometer system. Thermogravimetric analysis (TGA) was carried out on a Netzsch STA 449 F3 Jupiter simultaneous thermal analyzer. The ratio of the ITO and RGO was determined by TGA measured from 30 to 800 °C at a heating rate of 10 °C/min in air. High resolution transmission electron microscopy (HRTEM) samples were prepared by ultrasonically dispersing the anode materials in ethanol and drying them on Holey carbon/copper grids. These samples were observed under a JEOL 2010 FasTEM with 200 kV thermionic electron source. Energy-dispersive X-ray spectroscopy (EDS) was used to estimate the concentration of In atoms in the ITO crystals. X-ray photoelectron spectroscopy (XPS) analysis was performed using a PHI multiprobe system with twin anode XPS using unmonochromatized Al K% radiation (1486.6 eV) operated at 250 W and 15 kV. The pressure in the analysis chamber was always ~10^−8^ Torr or less. The full survey was taken at 100 eV pass energy with a scan rate of 1 eV/s and the high resolution scans were conducted at a 20 eV pass energy with a scan rate of 0.1 eV/s. The spectra were calibrated with respect to graphitic C 1 s at 284.6 eV. The background was determined using the Shirley-type background correction and the curves were fitted with Gaussian/Lorentzian product functions.

### Anode Fabrication and Coin Cell Fabrication

Anodes were fabricated by preparing inks containing 80 wt% active material, 10 wt% carbon black (Vulcan XC-72R, Cabot) as the conducting agent, and 10 wt% binder, made by dispersing polyvinylidene fluoride (PVDF, Kynar blend) in N-methylpyrrolidone (NMP, Acros, 99.5% Extra Dry). The inks were homogenized through repeated and successive sonication and stirring, coated onto a copper foil (Alfa Aesar, 99.999%), dried at 100 °C under vacuum for 12 h, then pressed at 1500 lbs for 5 min. For all electrodes fabricated in this study, the active loading was 0.2–0.8 mg active material/cm^2^. Coin cells were assembled to test the electrochemical properties of anodes in a half-cell configuration. Coin cells (2 cm in diameter, Hohsen Corp.) were assembled in an argon-filled glovebox (Labconco) with the anodes as the working electrode, lithium metal (Alfa Aesar, 99.9%) as both the counter and reference electrode, Celgard 2320 tri-layer PP/PE/PP as the separator, and a mixture of 1 M lithium hexafluorophosphate (LiPF_6_, Acros 98%) in (1:1:1 vol) ethylene carbonate (EC, Acros 99+%):dimethyl carbonate (DMC, Acros 98+%):diethyl carbonate (DEC, Acros 99%) as the electrolyte.

### Electrochemical Testing

All charge-discharge C-rate calculations were based on the SnO_2_ conversion reaction theoretical capacity of 780 mAh/g. Charge-discharge measurements were carried out at various C-rates between 0.001 and 3 V (vs. Li^+^/Li) using an Arbin MSTAT battery test system. Cyclic Voltammograms (CV) were collected at a scan rate of 0.1 mV/s over the same voltage windows as the charge/discharge cycles. Electrochemical Impedance Spectroscopy (EIS) was conducted between 100 kHz–50 mHz with a 5 mV amplitude at the coin cell open circuit voltage, using an Autolab PGSTAT302N Potentiostat (Eco Chemie).

### Identical-Location TEM

The identical-location transmission electron microscopy (IL-TEM) samples were prepared by spraying the ITO/RGO ink containing 10% PVDF binder on a 3 mm diameter copper TEM finder grid (Ted Pella, Inc., 100 mesh). The grid was then dried on a hot plate at 210 °C for 3 min. TEM images of the ITO/RGO at the same position of the TEM finder grid were collected using a novel experimental setup detailed in our previous paper^21^. Briefly, after taking the TEM images of the fresh ITO/RGO, the copper grid was placed in a custom-built Teflon-shrouded copper electrode with a Teflon cap. Pressure was applied by the hollow cap to ensure the contact between grid and Cu electrode. The hollow structure of the cap facilitated the electrolyte access to the working electrode. To complete the electrochemical cell, a strip of Celgard was sandwiched between a piece of lithium metal and the working electrode. The entire assembly was submerged into a EC:DMC:DEC (1:1:1) electrolyte. The CVs were collected between 0.001–3 V at 0.1 mV/s, then the electrode/TEM grid setup was taken out and dried without rinsing and imaged.

## Additional Information

**How to cite this article**: Liu, Y. *et al*. Highly Conductive In-SnO_2_/RGO Nano-Heterostructures with Improved Lithium-Ion Battery Performance. *Sci. Rep*. **6**, 25860; doi: 10.1038/srep25860 (2016).

## Supplementary Material

Supplementary Information

## Figures and Tables

**Table 1:** Impedance parameters (Ω) of anodes before and after 600 cycles.

Anodes	R1	R2+R3
SnO_2_/RGO before	11.21	27.47
SnO_2_/RGO after	10.96	44.4
ITO/RGO before	10.6	21.87
ITO/RGO after	11.1	21.71
