# Supplementary material for: Highly Conductive In-SnO2/RGO Nano-Heterostructures with Improved Lithium-Ion Battery Performance
Source: Sci Rep. 2016 May 11;6:25860. doi: 10.1038/srep25860 (PMC4863165; doi:10.1038/srep25860)
Supplement: Supplementary Information [file srep25860-s1.pdf]

Highly Conductive In-SnO_2/RGO Nano-Heterostructures with Improved Lithium-Ion Battery Performance

Ying Liu[1,2], Alessandro Palmieri[1,2], Junkai He[3,4], Yongtao Meng[3,4], Nicole Beauregard[1,2], Steven L. Suib[3,4] and William E. Mustain[1,2,3†]

1 Department of Chemical & Biomolecular Engineering, University of Connecticut Storrs, CT 06269-3222

2 Center for Clean Energy Engineering, University of Connecticut, Storrs, CT 06269-5233

3 Institute of Materials Science, University of Connecticut, Storrs, CT 06269-3136

4 Department of Chemistry, University of Connecticut, Storrs, CT

† Email: mustain@engr.uconn.edu

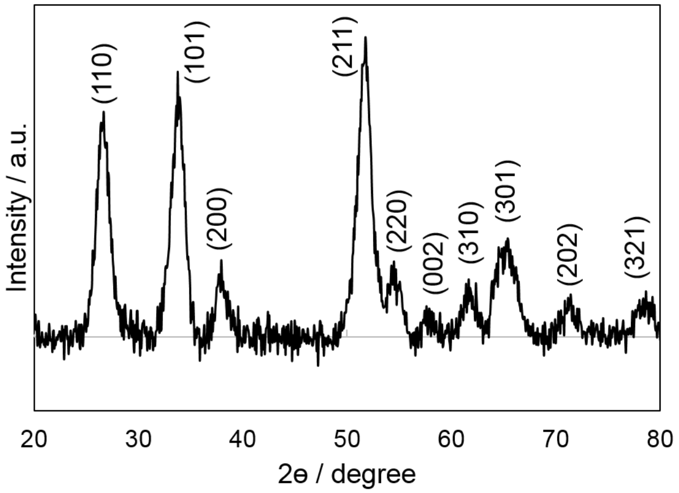

Figure S1. XRD pattern of ITO prepared without graphene sheets. All of the diffraction peaks are ascribed to the pure phase of well-crystalized SnO_2 (JCPDS 041-1445).

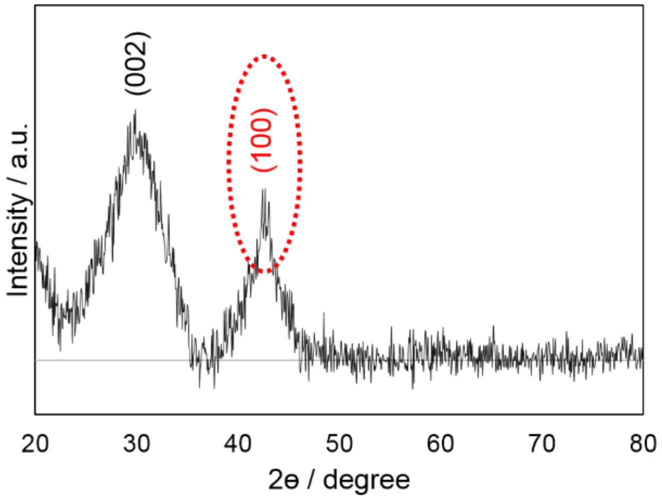

Figure S2. XRD pattern of the as-prepared graphene sheets after exfoliation.

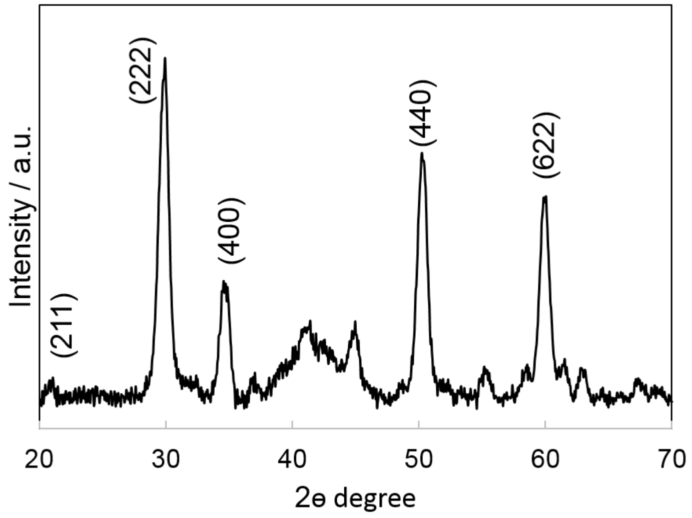

Figure S3. XRD pattern of the In_2O_3/RGO composite, shows a bixbyite In_2O_3 cubic structure (JCPDS 06-0416).

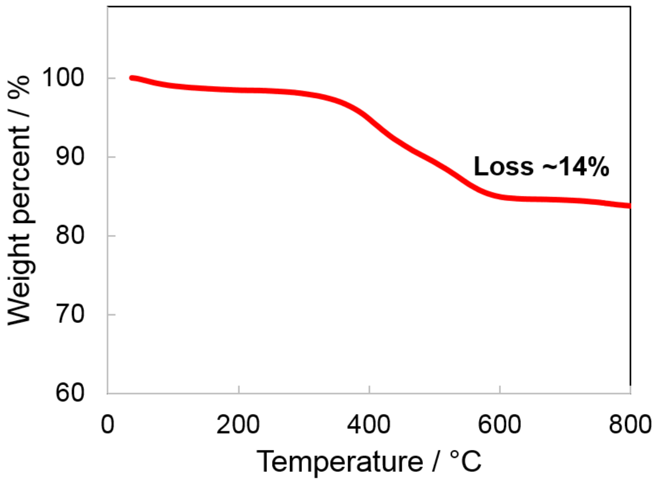

Figure S4. TGA curves of ITO/RGO composite measured from 30 to 800 ℃ at a heating rate of 10 ℃/min in air. The weight percentage of graphene sheets in the ITO/RGO composites was estimated to be ~14 wt%.

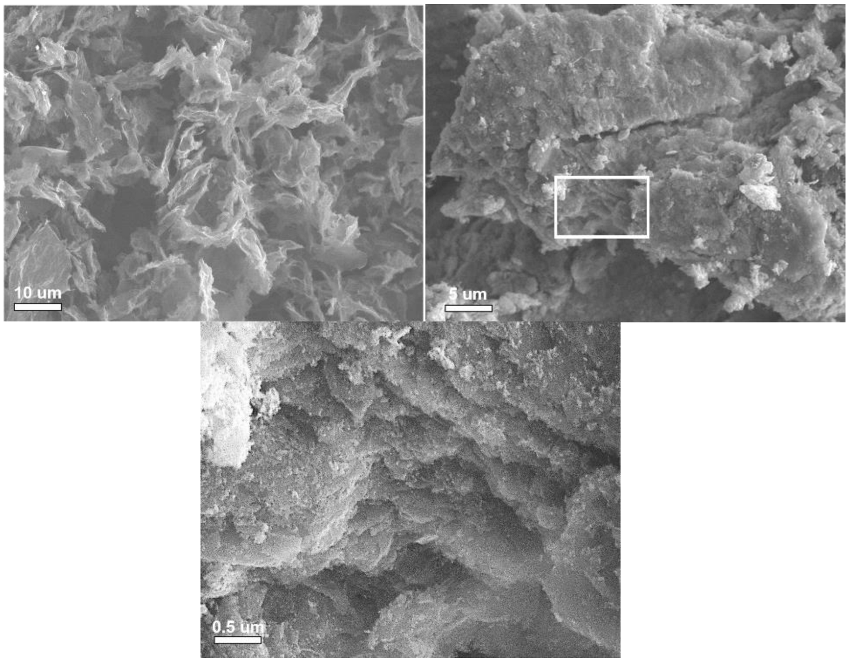

Figure S5. SEM images of (a) graphene and (b) (c) ITO/RGO.

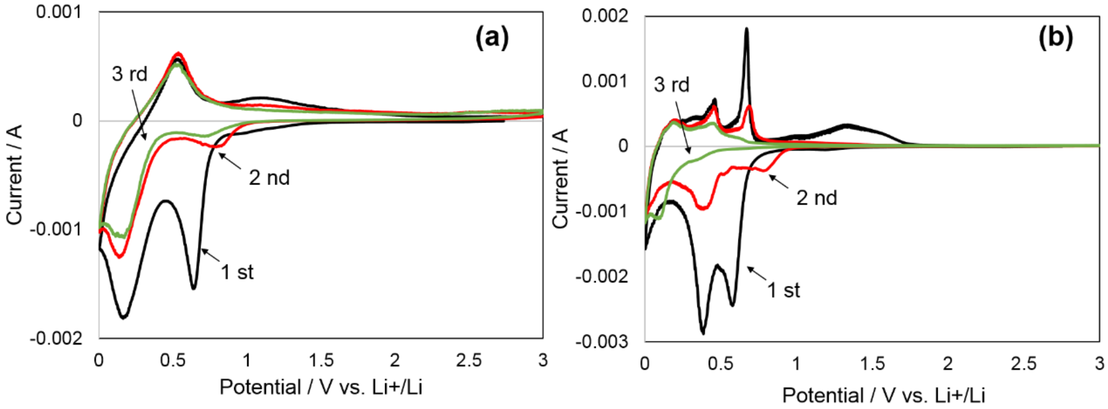

Figure S6. Cyclic voltammograms (CV) of the (a) SnO_2/RGO and (b) In_2O_3/RGO composite between 0.01 and 3V vs. Li^+/Li at a scan rate of 0.1 mV/s.

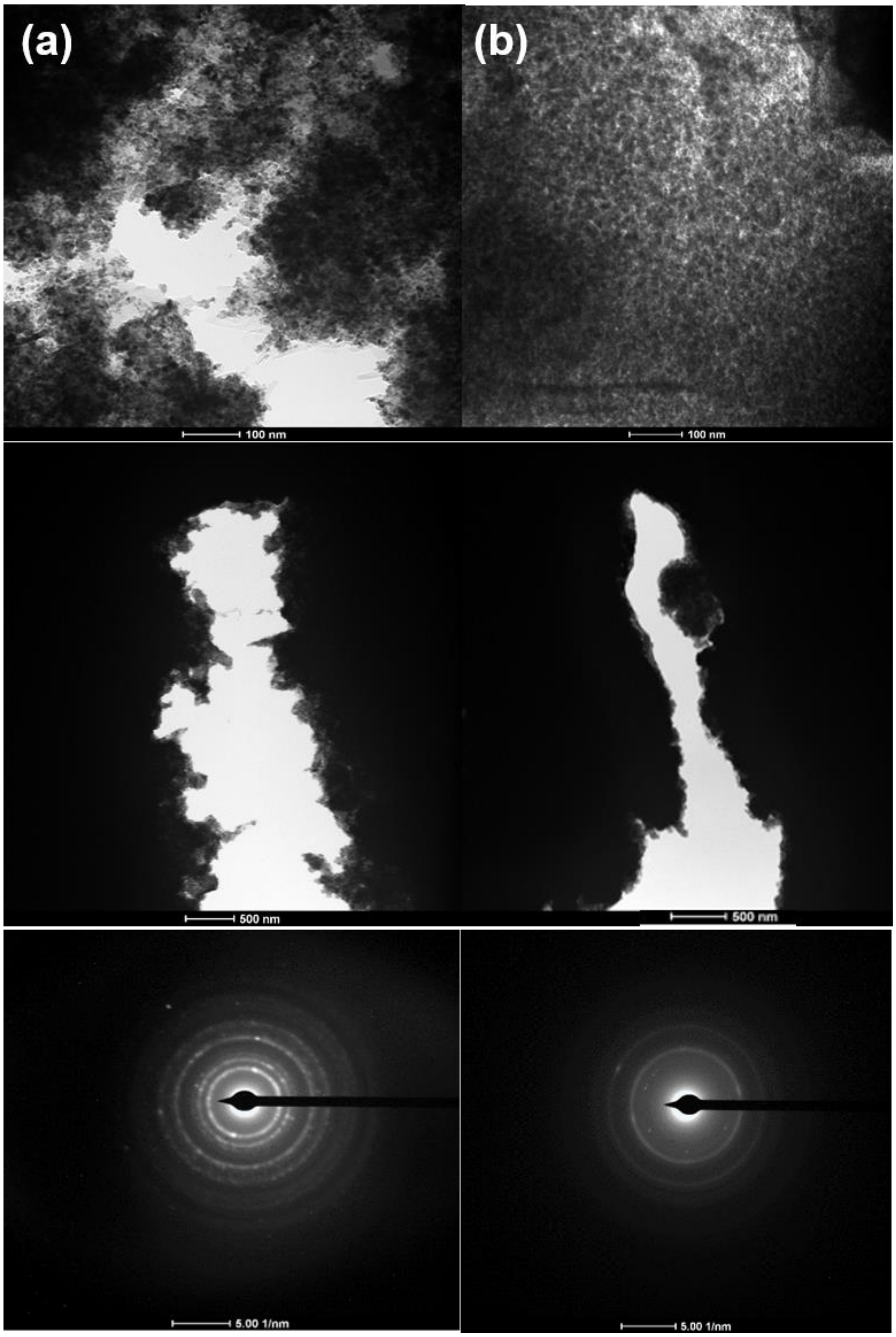

Figure S7. IL-TEM images and diffraction patterns for ITO/RGO on a Cu TEM grid (a) before cycling, (b) after two cycles. Cyclic voltammetry was performed at 0.1 mV/s between 0.001 and 3 V vs. Li/Li^+ in a 1M $LiPF_6$ in (1:1:1) EC-DMC-DEC electrolyte.
